# Estatísticas Cardiovasculares do Programa Boas Práticas em Cardiologia – Dados de um Hospital Público Terciário Brasileiro

**DOI:** 10.36660/abc.20220247

**Published:** 2023-02-10

**Authors:** Luiz Guilherme Passaglia, Marina Lírio Resende Cerqueira, Mariana Martins Pires, Lucas Vieira Chagas, Carolina Teixeira Cunha, Erika Nunes de Oliveira Rodrigues, Flávia Mariana Mendes Diniz, Darkiane Fernandes Ferreira, Monique Rocha Nogueira, Gisia Teodoro Braga, Fábio P. Taniguchi, Antonio Luiz Pinho Ribeiro

**Affiliations:** 1 Universidade Federal de Minas Gerais Serviço de Cardiologia e Cirurgia Cardíaca Hospital das Clínicas Belo Horizonte MG Brasil Universidade Federal de Minas Gerais – Serviço de Cardiologia e Cirurgia Cardíaca do Hospital das Clínicas, Belo Horizonte, MG – Brasil; 2 Universidade Federal de Minas Gerais Faculdade de Medicina Belo Horizonte MG Brasil Universidade Federal de Minas Gerais – Faculdade de Medicina, Belo Horizonte, MG – Brasil; 3 Universidade Federal de Minas Gerais Equipe de Enfermagem Hospital das Clínicas Belo Horizonte MG Brasil Universidade Federal de Minas Gerais – Equipe de Enfermagem do Hospital das Clínicas, Belo Horizonte, MG – Brasil; 4 Hospital do Coração São Paulo SP Brasil Hospital do Coração (Hcor), São Paulo, SP – Brasil; 5 Universidade Federal de Minas Gerais Departamento de Clínica Médica Hospital das Clínicas Belo Horizonte MG Brasil Universidade Federal de Minas Gerais – Departamento de Clínica Médica da UFMG e Centro de Telessáude do Hospital das Clínicas,Belo Horizonte, MG – Brasil

**Keywords:** Doenças Cardiovasculares, Síndrome Coronariana Aguda, Insuficiência Cardíaca, Melhoria de Qualidade

## Abstract

**Fundamento:**

O Programa Boas Práticas em Cardiologia é uma iniciativa da Sociedade Brasileira de Cardiologia (SBC) destinada à melhoria do cuidado cardiovascular nos hospitais públicos brasileiros.

**Objetivos:**

Descrever características dos pacientes internados com Síndrome Coronariana Aguda (SCA) e Insuficiência Cardíaca (IC) e avaliar os indicadores de desempenho alcançados nos braços (SCA e IC) em um hospital público terciário, com uma meta pré-estabelecida de 85% de aderência às recomendações da SBC.

**Métodos:**

Estudo do tipo transversal descritivo realizado por meio da coleta de dados de pacientes que estiveram internados entre maio de 2016 e setembro de 2019.

**Resultados:**

Foram incluídos 1036 pacientes, 273 pacientes no braço IC e 763 no braço SCA. A média de idade foi de 59,8 ± 12,0 anos na SCA e 57,0 ± 14,1 anos na IC, com predomínio do sexo masculino em ambos os grupos. Mais da metade dos pacientes não tinham ensino fundamental completo e mais de 90% declararam renda mensal inferior a cinco salários-mínimos. Na SCA, predominou o diagnóstico de SCA com supradesnivelamento do segmento ST (66,3%) e 2,9% dos pacientes foram a óbito. Na IC, a etiologia mais comum foi a Doença de Chagas (25,8%) e 17,9% dos pacientes foram a óbito. Na avaliação dos indicadores de desempenho, nove dos 12 indicadores tiveram taxas de aderência acima de 85%.

**Conclusão:**

Programas de qualidade são essenciais à melhoria do cuidado e os indicadores de desempenho do hospital apontam para uma boa adesão às diretrizes assistenciais da SBC, particularmente no braço da SCA.

## Introdução

As doenças cardiovasculares (DCV) constituem a principal causa de morte no mundo e uma das cinco principais causas de anos de vida saudáveis perdidos.^1^ No Brasil, a síndrome coronariana aguda (SCA) é a principal condição cardíaca que leva ao óbito e a insuficiência cardíaca (IC) a principal condição cardíaca que leva à internação hospitalar.^2^

Organizações nacionais e internacionais têm desenvolvido e disseminado orientações por meio de diretrizes clínicas para auxiliar os profissionais de saúde no manejo das DCV.^3 - 5^ Apesar de uma vasta publicação de terapias comprovadamente eficazes, a adesão às recomendações disponíveis nas diretrizes ainda permanece abaixo do ideal,^6^ particularmente nos hospitais brasileiros, como demonstram os Registros ACCEPT,^7^ BRACE^8^ e BREATHE.^9^

Nesse contexto, programas de melhoria da qualidade assistencial foram desenvolvidos na tentativa de garantir um cuidado adequado aos pacientes com DCV. Nos Estados Unidos da América, o Programa *Get With the Guidelines (* Programa *GWTG)* é uma iniciativa criada pela *American Heart Association (AHA)* e pela *American Stroke Association* com a finalidade de melhorar a qualidade do atendimento hospitalar desses pacientes.^10^ No Brasil, a Sociedade Brasileira de Cardiologia (SBC) e o Ministério da Saúde, junto do AHA e em colaboração com o Hospital do Coração de São Paulo - Hcor criaram o Programa Boas Práticas em Cardiologia (Programa BPC). Esse programa tem a finalidade de avaliar a taxa de adesão às recomendações das diretrizes da SBC, assim como avaliar o resultado da implementação de um programa de qualidade nos desfechos clínicos dos pacientes internados por DCV.^11^

Diante disso, o presente estudo tem como objetivo caracterizar dados da internação de pacientes com SCA e IC, participantes do Programa BPC, em um hospital público terciário brasileiro, assim como avaliar as taxas de adesão às terapias baseadas em evidências, determinadas pela SBC e definidas como indicadores de desempenho.

## Métodos

O presente estudo é do tipo transversal descritivo realizado por meio da coleta de dados de pacientes internados no Hospital das Clínicas da Universidade Federal de Minas Gerais entre maio de 2016 e setembro de 2019 com diagnóstico primário de IC ou SCA. Detalhes do desenho e fundamento do Programa BPC foram previamente publicados,^11^ e os braços realizados nesse hospital tiveram aprovação do Comitê de Ética Médica da Universidade Federal de Minas Gerais (Número: 1.487.029 de 11/04/2016).

### População

Foram incluídos pacientes com idade igual ou superior a 18 anos, admitidos com diagnóstico primário de SCA (CID-10 códigos I20.0 a I21.9 e I22.0 a I22.9) ou IC (CID-10 códigos I50.0; I50.1 ou I50.9), independente de história prévia dessas condições. Os critérios de exclusão foram:

(A) Pacientes com SCA desencadeada por revascularização miocárdica eletiva (intervenção coronária percutânea eletiva ou cirurgia de revascularização do miocárdio) ou por procedimentos cirúrgicos maiores, não cardíacos, durante internação hospitalar.

(B) Pacientes com história prévia de IC, mas que foram admitidos na sala de emergência com diagnóstico clínico confirmado de dispneia por outras causas. Também foram excluídos pacientes com internação por IC e com remoção planejada para outro estabelecimento ou planejamento de internação hospitalar por menos de 24 horas.

### Coleta de Dados

Após a triagem quanto aos critérios de elegibilidade, os pacientes eram questionados se desejavam participar do programa BPC ainda durante a internação, e o Termo de Consentimento Livre e Esclarecido foi assinado por todos os participantes antes da alocação. A coleta dos dados foi realizada a partir do prontuário médico e de entrevista presencial e estruturada por uma equipe treinada, utilizando os formulários desenvolvidos pelo Programa BPC.^11^

### Desfechos

O desfecho primário do Programa BPC foram as taxas de adesão da equipe assistencial aos indicadores de desempenho, que são métricas de qualidade determinadas a partir de recomendações classe I das diretrizes da SBC e AHA, descritas em detalhes em publicação anterior^11^ e no site do Programa BPC (http://www.cardiol.br/boaspraticasclinicas/).

Os indicadores de desempenho no braço de SCA foram (Suplemento 1):

1. Aspirina precoce;
2. Terapia de reperfusão adequada (tempo porta-agulha e tempo porta-balão);
3. Aspirina na alta;
4. Inibidor da Enzima de Conversão de Angiotensina (IECA) ou Bloqueador do Receptor de Angiotensina (BRA) na alta em pacientes com Fração de Ejeção do Ventrículo Esquerdo (FEVE) < 45%;
5. Betabloqueador na alta;
6. Estatina na alta;
7. Controle da Pressão Arterial na alta;
8. Aconselhamento para parar de fumar para tabagistas ativos na alta.

Os indicadores de desempenho no braço de IC foram ( Suplemento 1 ):

1. Avaliação da função ventricular esquerda pelo ecocardiograma;
2. IECA ou BRA na alta;
3. Betabloqueador na alta;
4. Espironolactona na alta;
5. Consulta pós-alta (acompanhamento ambulatorial pré-agendado na alta hospitalar).

Para o programa BPC, foi estabelecido como meta um limiar mínimo de aderência de 85% para os indicadores de desempenho descritos acima com base em resultados prévios do programa *GWTG.* ^12^

### Análise estatística

Por ser um estudo observacional descritivo com uma amostra de conveniência, não foi realizado o cálculo amostral. As variáveis contínuas foram resumidas como média e desvio padrão ou como mediana e primeiro e terceiro quartis (Q1, Q3) conforme resultado do teste de normalidade (Shapiro-Wilk), considerando o nível de significância de 5%. As variáveis categóricas foram expressas como proporções. Para o denominador de cada análise, foram considerados apenas os dados válidos da variável coletada, excluindo, portanto, os pacientes com dados incompletos e os pacientes que apresentavam contraindicações ao uso dos medicamentos que compõem os indicadores de desempenho, registradas no prontuário médico. Os dados foram analisados com a utilização do programa SPSS para Windows, versão 20.1 (IBM Corp, Armonk, NY, EUA).

## Resultados

Neste estudo foram incluídos 1036 pacientes, sendo 763 pacientes com diagnóstico de SCA (“braço de SCA”) e 273 pacientes com diagnóstico de IC (“braço de IC”). A média de idade foi de 59,8 ± 12,0 anos nos pacientes do braço de SCA e 57,0 ± 14,1 anos nos pacientes do braço de IC, com predomínio do sexo masculino em ambos.

As principais comorbidades dos pacientes foram hipertensão, diabetes e dislipidemia. No braço de IC houve uma grande proporção de pacientes com hipotireoidismo e outras doenças de base. Uma significativa parcela dos pacientes declarou tabagismo atual ou passado, com destaque para o tabagismo atual no braço de SCA ( Tabela 1 ).

**Tabela 1:** – Caracterização da amostra na admissão hospitalar (dados gerais e histórico médico)

Variável		Síndrome Coronariana Aguda (763)	Insuficiência Cardíaca (273)
Idade, m±dp*		59,8 ± 12,0	57,0 ± 14,1
Sexo Feminino		225/763 (29,5%)	119/273 (43,6%)
Comorbidades	Hipertensão arterial sistêmica	497/633 (78,5%)	108/260 (41,5%)
	Diabetes mellitus	220/633 (34,8%)	69/260 (26,5%)
	Dislipidemia	171/633 (27,0%)	38/260 (14,6%)
	Acidente vascular encefálico ou acidente isquêmico transitório	49/633 (7,7%)	18/260 (12,3%)
	Doença arterial periférica	38/633 (6,0%)	17/260 (6,5%)
	Hipotireoidismo	35/633 (5,5%)	41/260 (15,8%)
	Doença pulmonar obstrutiva crônica/asma	47/633 (7,4%)	29/260 (11,2%)
Cardiopatia prévia	Doença coronariana	162/633 (25,6%)	29/260 (11,2%)
	Infarto agudo do miocárdio	119/633 (18,8%)	44/260 (16,9%)
	Insuficiência cardíaca	30/633 (4,7%)	236/260 (90,8%)
	Fibrilação ou Flutter atrial	21/633 (3,3%)	99/260 (38,1%)
	Doença valvar	8/633 (1,3%)	39/264 (15,2%)
	Doença de Chagas	6/633 (0,8%)	66/264 (25,8%)
Procedimentos Cardiológicos prévios	Angioplastia coronariana	98/763 (12,8%)	31/273 (11,4%)
	Cirurgia de revascularização miocárdica	39/763 (5,1%)	8/273 (2,9%)
	Marcapasso	6/763 (0,8%)	43/273 (15,8%)
	Ressincronizador	2/763 (0,3%)	4/273 (1,5%)
	cardiodesfibrilador	5/763 (0,7%)	39/273 (14,3%)
	Transplante cardíaco	-	9/273 (6,3%)
Tabagismo	Total (atual e prévio)	509/761 (66,8%)	139/266 (55,3%)
	Atual	253/761 (33,2%)	21/266 (7,9%)
	Prévio	256/761 (33,6%)	118/266 (44,4%)
	Não	252/761 (33,1%)	127/266 (47,7%)

* Variável apresentada como média ± desvio padrão (m±dp).

Mais da metade dos pacientes incluídos no estudo não tinham ensino fundamental completo e mais de 90% declararam renda mensal inferior a cinco salários-mínimos ( Tabela 2 ).

**Tabela 2:** – Caracterização das variáveis socioeconômicas

Variável		Síndrome Coronariana Aguda (763)	Insuficiência Cardíaca (273)
Grau de Escolaridade	Não alfabetizado	72/761 (9,5%)	29/268 (10,8%)
	Fundamental incompleto	372/761 (48,9%)	127/268 (47,4%)
	Fundamental completo	91/761 (12,0%)	38/268 (14,2%)
	Ensino médio incompleto	67/761 (8,8%)	12/268 (4,5%)
	Ensino médio completo	85/761 (11,2%)	52/268 (19,4%)
	Ensino superior incompleto	34/761 (4,5%)	5/268 (1,9%)
	Ensino superior completo	40/761 (5,3%)	5/268 (1,9%)
Renda Familiar	≤ 1 salário-mínimo	192/761 (25,2%)	76/268 (28,4%)
	> 1 a ≤ 2 salários-mínimos	276/761 (36,3%)	131/268 (48,9%)
	> 2 a ≤ 5 salários-mínimos	226/761 (29,7%)	46/268 (17,2%)
	> 5 a ≤ 10 salários-mínimos	47/761 (6,2%)	12/268 (4,5%)
	> 10 salários-mínimos	20/761 (2,6%)	3/268 (1,1%)

No braço de SCA ( Tabela 3 ), a maior parte dos pacientes foram admitidos em classificação de Killip-Kimball I-II (81,6%) e mais de um terço (37,4%) apresentou recorrência de dor torácica nas primeiras 24 horas da internação hospitalar. Predominou o diagnóstico de infarto agudo do miocárdio (IAM) com supradesnivelamento do segmento ST (66,3%), sendo que 42,9% desses pacientes foram trombolisados, 33,8% realizaram angioplastia primária e 23,3% não foram submetidos a nenhuma terapia de reperfusão.

**Tabela 3:** – Caracterização dos pacientes com síndrome coronariana aguda no momento da internação

Tipo de síndrome coronariana aguda	Angina instável		94/758 (12,4%)
	IAM sem supra de ST		156/758 (20,6%)
	IAM com supra de ST		506/758 (66,7%)
Classificação de Killip-Kimball	Killip I		491/755 (65,0%)
	Killip II		125/755 (16,5%)
	Killip III		55/755 (7,2%)
	Killip IV		58/755 (7,7%)
Parada cardiorrespiratória à admissão			59/759 (7,8%)
Recorrência de dor nas primeiras 24h			220/589 (37,4%)
Exames laboratoriais à admissão*	Creatinina, mg/dL - m±dp		0,96 ± 0,2
	Colesterol Total, mg/dL - md (IIQ)		168 (140-202)
	LDL-C, mg/dL - md (IIQ)		98 (72-129)
	HDL-C, mg/dL - md (IIQ)		41 (34-49)
	Triglicerídeos, mg/dL - md (IIQ)		112 (76-170)
Candidatos a reperfusão			506/757 (67,1%)
Terapia de reperfusão química	Trombolítico		217/506 (42,9%)
	Tipo de Trombolítico	Estreptoquinase	1/217 (0,5%)
		Alteplase	175/217 (80,6%)
		Não documentado	41/217 (18,9%)
Cineangiocoronariografia	Cineangiocoronariografia diagnóstica		706/757 (93,3%)
	Realizado ICP do vaso culpado		495/756 (65,5%)
	ICP primária		171/506 (33,8%)
	ICP de resgate		84/495 (17,0%)
Cineangiocoronariografia (localização das lesões)	Sem lesões		45/547 (8,6%)
	Univascular		150/547 (27,4%)
	Bivascular		145/547 (26,5%)
	Trivascular		207/547 (37,8%)
	Lesão de TCE		21/547 (3,8%)
	Lesão de DA proximal		187/547 (34,2%)
Fração de ejeção do ventrículo esquerdo, % - md (IIQ)*			54 (43-62)
Cirurgia de revascularização miocárdica			43/763 (5,6%)
Número de dias em terapia intensiva – md (IIQ)*			4 (3-6)

DA: artéria descendente anterior; HDL-C: high-density lipoprotein cholesterol; IAM: infarto agudo do miocárdio; ICP: intervenção coronária percutânea; LDL-C: low-density lipoprotein cholesterol; TCE: tronco de coronária esquerda . * Variáveis apresentadas como média ± desvio padrão (m±dp) ou mediana e 1º e 3º quartis [md (IIQ)].

No braço de IC ( Tabela 4 e Suplemento 2 ), as etiologias mais comuns foram a Doença de Chagas (25,8%), a forma idiopática (22,3%), a cardiopatia isquêmica (15,2%) e a doença valvar (15,2%). A maioria dos pacientes internou em classe funcional de NYHA ( *New York Heart Association* ) III-IV (76,4%), com predomínio do perfil hemodinâmico “quente e úmido” (60,1%) à admissão. A FEVE média da amostra foi de 35,0% ± 9,0% e 50 pacientes (18,3%) foram encaminhados na mesma internação hospitalar para a realização de transplante cardíaco. Ao longo da internação, a dobutamina foi usada em algum momento em 53,9% dos pacientes, ou por admissão já no perfil “frio”, ou por evolução para sinais de baixo débito cardíaco, assim como a furosemida venosa em “bolus” foi usada em 90,2% dos pacientes e a furosemida contínua na bomba de infusão em 25,4%.

**Tabela 4:** – Caracterização dos pacientes com insuficiência cardíaca no momento da internação

Etiologia da insuficiência cardíaca	Chagas	66/264 (25,8%)
	Idiopática	57/264 (22,3%)
	Isquêmica	39/264 (15,2%)
	Valvar	39/264 (15,2%)
	Hipertrófica	8/264 (3,1%)
	Cardiotoxicidade	5/264 (2,0%)
	Hipertensiva	5/264 (2,0%)
	Outras	45/264 (17,0%)
Número de internações nos últimos 6 meses	0	44/184 (23,9%)
	1	40/184 (21,7%)
	2	42/184 (22,8%)
	> 2	58/184 (31,5%)
Classe Funcional de *New York Heart Association (NYHA)*	I	3/254 (1,2%)
	II	16/254 (5,9%)
	III	70/254 (27,6%)
	IV	124/254 (48,8%)
Paciente na fila do transplante cardíaco	Sim	70/259 (27%)
	Não	189/259 (73%)
Perfil hemodinâmico	Quente e seco	8/253 (3,2%)
	Quente e úmido	152/253 (60,1%)
	Frio e úmido	62/253 (24,5%)
	Frio e seco	8/253 (3,2%)
Causa da descompensação	Não adesão medicamentosa	23/273 (8,4%)
	Não adesão alimentar ou hídrica	11/273 (4,0%)
	Hipertensão não controlada	5/273 (1,8%)
	Infecção	42/273 (15,4%)
	Arritmia	46/273 (16,8%)
	Descompensação da doença renal	11/273 (4,0%)
	Isquemia ou Síndrome Coronariana Aguda	14/273 (5,1%)
Dados do ecocardiograma*	Fração de Ejeção do Ventrículo Esquerdo, % - m±dp	35,0 ± 9,0
	Diâmetro do Átrio Esquerdo, milímetros - m±dp	49,5 ± 9,0
Outros dados da internação	Profilaxia para Trombose Venosa Profunda	237/256 (92,6%)
	Registro de peso (pelo menos 70% do período da internação)	173/256 (67,8%)
Suspensão do betabloqueador (se uso prévio), na admissão ou internação	Sim	81/141 (57,4%)
	Não	60/141 (42,6%)

* Dados completos disponíveis no sistema (denominador da variável) - dados do ecocardiograma (217 pacientes). Variável apresentada como média ± desvio padrão (m±dp)

As Tabelas 5 e 6 mostram os dados de mortalidade intra-hospitalar e dados da alta hospitalar. Foram a óbito na internação 2,9% dos pacientes no braço de SCA e 17,9% dos pacientes no braço IC.

**Tabela 5:** – Dados dos pacientes com síndrome coronariana aguda no momento da alta

Óbitos intra-hospitalar				22/763 (2,9%)
Medicamentos na alta		Ácido acetilsalicílico		700/733 (95,5%)
		Inibidor da P2Y12		657/733 (89,6%)
		Anticoagulante		94/733 (12,8%)
		Betabloqueador		616/657 (93,7%)
				BB contraindicado em 76/733 (10,4%)
		Inibidor da Enzima de Conversão de Angiotensina		439/596 (73,7%)
				IECA contraindicado em 137/733 (18,6%)
		Bloqueador do receptor de angiotensina (BRA)		142/635 (22,4%)
				BRA contraindicado em 98/733 (13,4%)
		Estatina		707/733 (96,5%)
		Espironolactona		78/486 (16,0%)
				Espironolactona contraindicada em 247/733 (33,7%)
Dados clínicos na alta*		PA sistólica, mmHg – md (IIQ)		113 (100-130)
		PA diastólica, mmHg – md (IIQ)		70 (60-80)
		FC, batimentos por minuto – md (IIQ)		68 (63-78)
Orientações	Cessação de Tabagismo^†^	Sim		277/340 (81,4%)
		Não		3/340 (0,9%)
		Não se aplica		393/733 (53,6%)
		Não documentado		60/340 (17,6%)
	Mudança de estilo de vida	Sim		670/733 (91,4%)
		Não		16/733 (2,2%)
		Não documentado		46/733 (6,3%)
	Controle do peso	Sim		546/710 (76,9%)
		Não		24/710 (3,3%)
		Não se aplica		23/733 (3,1%)
		Não documentado		140/710 (19,7%)
	Exercício físico	Sim		660/733 (90,0%)
		Não		17/733 (2,3%)
		Não documentado		56/733 (7,7%)
	Referenciado à Reabilitação^†^	Sim		615/721 (85,2%)
		Não		32/721 (4,4%)
		Não se aplica		12/733 (1,6%)
		Não documentado		74/721 (10,3%)
	Uso dos medicamentos prescritos	Sim		677/733 (92,4%)
		Não		9/733 (1,2%)
		Não documentado		47/733 (6,4%)
	Retorno ambulatorial	Sim		717/733 (97,8%)
		Não		4/733 (0,5%)
		Não se aplica		0/733 (0,0%)
		Não documentado		12/733 (1,7%)
	Terapia anticoagulante^†^		Sim	85/114 (74,6%)
			Não	14/114 (12,3%)
			Não se aplica	619/733 (84,4%)
			Não documentado	15/114 (13,1%)

FC: frequência cardíaca; PAD: pressão arterial diastólica; PAS: pressão arterial sistólica; AAS: ácido acetilsalisílico. * Variáveis apresentadas como mediana e 1º e 3º quartis [md (IQQ)]. † Cálculo feito em relação aos pacientes em que se aplica o tipo de orientação.

**Tabela 6:** – Dados dos pacientes com insuficiência cardíaca no momento da alta

Óbitos intra-hospitalar			49/273 (17,9%)
		Anticoagulante	93/206 (45,1%)
		BB	136/145 (93,8%)
			BB contraindicado em 61/206 (29,6%)
		IECA	72/128 (56,2%)
			IECA contraindicado em 78/206 (37,9%)
		BRA	48/145 (33,1%)
			BRA contraindicado em 61/206 (29,6%)
		Hidralazina	27/206 (13,1%)
		Nitrato	23/206 (11,2%)
		Antiarrítmicos	30/206 (14,6%)
		Digoxina	26/206 (12,6%)
		Diurético de Alça	156/206 (75,7%)
		Diurético Tiazídico	26/206 (12,6%)
		Espironolactona	88/124 (70,9%)
			Espironolactona contraindicada em 82/206 (39,8%)
Dados clínicos na alta*		PAS, mmHg - m±dp	107,1 ± 18,5
		PAD, mmHg - m±dp	67,3 ± 12,4
		FC, batimentos por minuto - m±dp	77,9 ± 14,2
Orientações	Cessação de tabagismo ^†^	Sim	10/27 (37,0%)
		Não	17/27 (63,0%)
		Não se aplica	180/207 (87,0%)
		Não documentado	0/27 (0,0%)
	Controle do peso	Sim	122/207 (58,9%)
		Não	8/207 (3,9%)
		Não documentado	77/207 (37,2%)
	Exercício físico	Sim	119/206 (57,8%)
		Não	16/206 (7,8%)
		Não documentado	71/206 (34,5%)
	Referenciado à reabilitação^†^	Sim	138/196 (70,4%)
		Não	5/196 (2,6%)
		Não se aplica	11/207 (5,3%)
		Não documentado	53/196 (27,0%)
	Uso dos medicamentos prescritos	Sim	180/206 (87,4%)
		Não	0/206 (0,0%)
		Não documentado	26/206 (12,6%)
	Aconselhamento em caso de piora dos sintomas	Sim	141/206 (68,4%)
		Não	3/206 (1,4%)
		Não documentado	62/206 (30,1%)
	Aconselhamento para vacinação (Influenza e Pneumococo)	Sim	57/207 (27,5%)
		Não	21/207 (10,1%)
		Não documentado	129/207 (62,3%)
	Retorno ambulatorial	Sim	202/207 (97,6%)
		Não	5/207 (2,4%)
		Não documentado	4/207 (1,9%)
	Orientações sobre terapia anticoagulante^†^	Sim	53/63 (84,1%)
		Não	1/63 (1,6%)
		Não se aplica	77/140 (55,0%)
		Não documentado	9/63 (14,3%)

BB: betabloqueador; IECA: inibidor da enzima de conversão de angiotensina; BRA: bloqueador do receptor de angiotensina; FC: frequência cardíaca; PAS: pressão arterial sistólica; PAD: pressão arterial diastólica; mmHg: milímetro de mercúrio. * Variáveis apresentadas como média ± desvio padrão (m±dp). † Cálculo feito em relação aos pacientes em que se aplica o tipo de orientação.

O Quadro 1 mostra os Indicadores de Desempenho analisados. É importante destacar que o indicador de “terapia de reperfusão adequada (tempo porta-agulha e tempo porta-balão)” não foi avaliado uma vez que o Hospital das Clínicas recebe pacientes referenciados de toda a rede pública metropolitana com o diagnóstico de SCA para a realização de cineangiocoronariografia, o que inviabiliza a contabilidade desses tempos. As taxas de adesão foram acima de 85% para seis dos sete indicadores analisados no braço de SCA e apenas para três dos cinco indicadores no braço de IC. A Figura Central resume os dados principais do artigo.

**Quadro 1:** – Indicadores de desempenho na alta hospitalar

	Indicadores de desempenho	
Síndrome Coronariana Aguda*	Aspirina precoce	739/756 (99,1%)
	Aspirina na alta hospitalar	700/733 (95,5%)
	IECA ou BRA na alta hospitalar	581/635 (91,5%)
	Betabloqueador na alta hospitalar	616/657 (93,7%)
	Estatina na alta hospitalar	707/733 (96,5%)
	Controle da pressão arterial	489/570 (85,8%)
	Aconselhamento para parar de fumar	277/340 (81,5%)
Insuficiência Cardíaca	Avaliação da função ventricular esquerda	242/243 (99,6%)
	IECA ou BRA na alta hospitalar	120/145 (82,7%)
	Betabloqueador na alta hospitalar	136/145 (93,8%)
	Espironolactona na alta hospitalar	88/124 (70,9%)
	Consulta pós-alta hospitalar	202/207 (97,6%)

IECA: inibidor da enzima de conversão de angiotensina; BRA: bloqueador do receptor de angiotensina. *Observação: o indicador de “terapia de reperfusão adequada” não foi avaliado, uma vez que o hospital é referência para angioplastia primária e todos os pacientes dessa amostra foram encaminhados ao serviço já com o diagnóstico de SCA.

**Figura Central:**
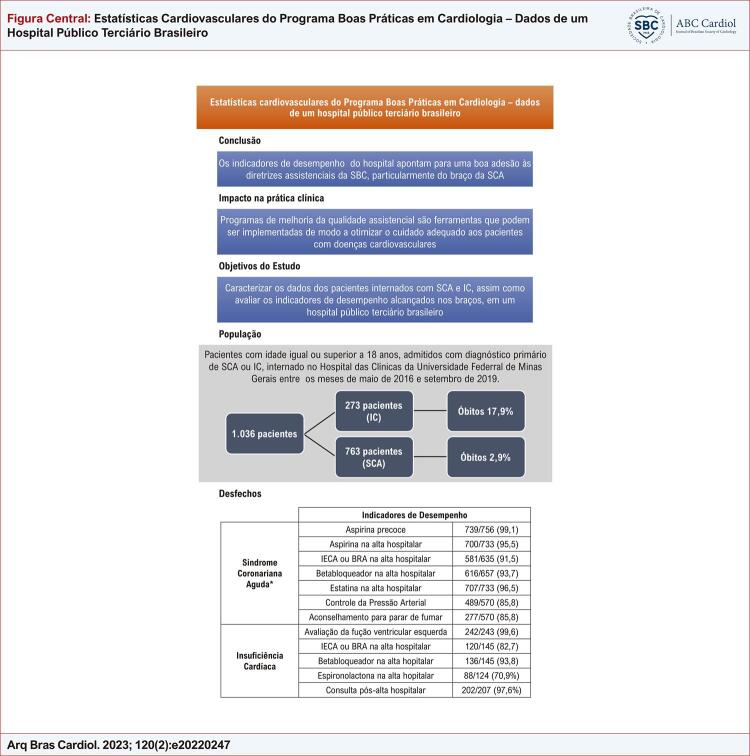
: Estatísticas Cardiovasculares do Programa Boas Práticas em Cardiologia – Dados de um Hospital Público Terciário Brasileiro

*IC: insuficiência cardíaca; IECA: inibidor da enzima de conversão de angiotensina; BRA: bloqueador do receptor de angiotensina; SCA: síndrome coronariana aguda; SBC: Sociedade Brasileira de Cardiogia.*

## Discussão

Os dados publicados nesse estudo caracterizam os pacientes internados por SCA e IC em um hospital federal, público e terciário, que possui todas as linhas de cuidado aos pacientes cardiopatas atendidos pelo Sistema Único de Saúde. Apesar da baixa renda e da alta taxa de pacientes não completamente alfabetizados, assim como uma alta complexidade clínica, essas condições não foram impeditivas para uma adequada adesão às terapias baseadas em evidência determinadas pela SBC, particularmente na linha de cuidado da SCA.

No Brasil, a SBC liderou a construção de registros clínicos das DCV, incluindo o registro nacional de SCA (estudo ACCEPT).^7^ Dados preliminares indicaram uma alta taxa de mortalidade intra-hospitalar, assim como baixas taxas de prescrições de terapias comprovadamente eficazes em pacientes internados. Esses dados são concordantes também com o Registro BRACE (Brazilian Registry on Acute Coronary Syndromes).^8^

Em comparação com esses dois registros,^7 , 8^ os pacientes internados com SCA no Hospital das Clínicas são um pouco mais jovens, mas com perfil semelhante de comorbidades, particularmente as altas taxas de hipertensão, diabetes e tabagismo. No entanto, duas importantes diferenças se fazem presente: em primeiro lugar, houve predomínio de IAM com supradesnivelamento do segmento ST, uma vez que o Hospital das Clínicas é um centro de referência da rede para angioplastia primária; e em segundo lugar, os indicadores de desempenho aqui apresentados no braço de SCA são quantitativamente melhores em comparação a todos os indicadores avaliados na alta hospitalar no BRACE.

Vale destacar que na cidade de Belo Horizonte, com o intuito de ampliar o acesso dos pacientes do Sistema Único de Saúde ao tratamento de SCA, foi implementada a linha de cuidado do IAM entre os anos de 2010 e 2011.^13^ Nesse processo, a participação do Hospital das Clínicas sempre se destacou por meio do Centro de Telessaúde, Unidade Coronariana, Serviço de Cirurgia Cardiovascular, Setor de Hemodinâmica, leitos de enfermaria especificamente dedicados a esses pacientes, além de ambulatórios para a continuidade do cuidado pós-alta hospitalar. Com isso, essa linha de cuidado já vinha sendo monitorada no hospital e, diante dos dados previamente publicados,^14^ o processo de educação continuada permitiu os resultados dos indicadores de desempenho apresentados no estudo atual.

Outro registro nacional também liderado pela SBC é o Registro BREATHE,^9^ da linha de cuidado da IC, que assim como para SCA encontrou uma baixa taxa de prescrição de medicamentos baseados na melhor evidência para essa doença. Quando comparados os dados do presente estudo com os do Registro BREATHE, os pacientes do Hospital das Clínicas apresentam diferenças em termos das principais etiologias da IC e da maior complexidade clínica. Isso se caracteriza pela presença de muitos pacientes em avaliação e/ou encaminhados para transplante cardíaco, pelo frequente uso de inotrópicos e vasodilatador venoso, assim como pela maior taxa de mortalidade intra-hospitalar. Análise dos indicadores de desempenho desse braço indica uma grande necessidade de melhorias na prescrição de alta e orientações ao paciente com IC.

Seguindo uma tendência mundial, os gastos com saúde pública em nosso país têm sido crescentes,^15^ e uma série de iniciativas podem ser desempenhadas com objetivo de melhorar a eficiência do sistema, particularmente iniciativas de melhoria da qualidade assistencial prestada aos pacientes com DCV.^16^ Foi com esse foco que a SBC criou o programa BPC^11^ e o Hospital das Clínicas implementou o programa em sua instituição. Nessa mesma linha, projetos como o uso de mensagens de texto por telefone celular para melhorar o controle dos fatores de risco cardiovasculares após alta hospitalar também foram desenvolvidos no hospital.^17 , 18^ Dados do Programa *GWTG* já são robustos e mostram que a adesão às diretrizes melhorou significativamente ao longo do tempo,^19^ gerando benefícios clínicos aos pacientes atendidos nas instituições ligadas a esse programa nos Estados Unidos da América.^20^ A SBC fortalece sua ação em prol de um melhor cuidado cardiovascular possível para todos os brasileiros.

Os dados do programa BPC no Hospital das Clínicas aqui apresentados possuem algumas limitações importantes. Em primeiro lugar, o número de dados faltantes foi elevado para algumas das variáveis, o que possivelmente interferiu na análise dos resultados. Em segundo lugar, por ser um estudo com necessidade de assinatura de termo de consentimento do paciente ou seus familiares, a mortalidade intra-hospitalar pode estar subestimada, pois pacientes graves que faleceram antes da assinatura do referido termo podem estar excluídos dessa amostra. Em terceiro lugar, a coleta de dados não pôde ser consecutiva ao longo de todo o período analisado, tanto devido a problemas relacionados à equipe da instituição envolvida com o programa BPC como por pequenos períodos de interrupção do próprio programa nacionalmente. Por fim, não foi possível apresentar o seguimento de 30 dias e seis meses após alta hospitalar devido ao número extremamente pequeno de dados registrados em sua completude.

Em contrapartida, a apresentação de dados nacionais, confiáveis e abrangentes sobre DCV é uma etapa obrigatória para a superação das desigualdades e para a oferta do melhor cuidado cardiovascular possível para todos os brasileiros. Este estudo reúne informações relevantes da SCA e IC de pacientes internados em um hospital terciário do Sistema Único de Saúde, e ajuda a preencher uma lacuna do conhecimento na literatura brasileira sobre o assunto, passo importante no planejamento da política de saúde no Brasil.

## Conclusão

A adesão aos programas de qualidade como o Programa BPC é um passo essencial à melhoria assistencial dos pacientes internados com SCA e IC. Os indicadores de desempenho alcançados apontam para uma boa adesão às diretrizes assistenciais da SBC, particularmente na linha de cuidado da SCA.

## * Material suplementar

Para informação adicional do Material Suplementar 1, por favor, clique aqui .

Para informação adicional do Material Suplementar 2, por favor, clique aqui .
