# Supplementary material for: Estatísticas Cardiovasculares do Programa Boas Práticas em Cardiologia – Dados de um Hospital Público Terciário Brasileiro
Source: Arq Bras Cardiol. 2023 Feb 10;120(2):e20220247. [Article in Portuguese] doi: 10.36660/abc.20220247 (PMC10263458; doi:10.36660/abc.20220247)
Supplement: Translation

# Translation: Cardiovascular Statistics from the Good Practices in Cardiology Program – Data from a Brazilian Tertiary Public Hospital

**Authors:** Luiz Guilherme Passaglia, Marina Lírio Resende Cerqueira, Mariana Martins Pires, Lucas Vieira Chagas, Carolina Teixeira Cunha, Erika Nunes de Oliveira Rodrigues, Flávia Mariana Mendes Diniz, Darkiane Fernandes Ferreira, Monique Rocha Nogueira, Gisia Teodoro Braga, Fábio P. Taniguchi, Antonio Luiz Pinho Ribeiro

**Affiliations:** 1 Brasil Universidade Federal de Minas Gerais – Serviço de Cardiologia e Cirurgia Cardíaca do Hospital das Clínicas, Belo Horizonte, MG – Brasil; 2 Brasil Universidade Federal de Minas Gerais – Faculdade de Medicina, Belo Horizonte, MG – Brasil; 3 Brasil Universidade Federal de Minas Gerais – Equipe de Enfermagem do Hospital das Clínicas, Belo Horizonte, MG – Brasil; 4 Brasil Hospital do Coração (Hcor), São Paulo, SP – Brasil; 5 Brasil Universidade Federal de Minas Gerais – Departamento de Clínica Médica da UFMG e Centro de Telessáude do Hospital das Clínicas,Belo Horizonte, MG – Brasil

**Keywords:** Cardiovascular Diseases, Acute Coronary Syndrome, Heart Failure, Quality Improvement

## Abstract

**Background:**

The Good Practices in Cardiology Program is an initiative created by the Brazilian Society of Cardiology (SBC) to improve the quality of care of cardiovascular disease patients in Brazilian public hospitals.

**Objectives:**

To characterize patients admitted to a tertiary public hospital with diagnosis of acute coronary syndrome (ACS) or heart failure (HF) and to evaluate performance indicators in both ACS and HF arms, with a pre-established target of 85% adherence to the SBC recommendations.

**Methods:**

This was a descriptive cross-sectional study through data collection of patients hospitalized between May 2016 and September 2019.

**Results:**

A total of 1,036 patients were included, 273 in the HF arm and 763 in the ACS arm. Mean age was 59.8 ± 12.0 years in the ACS and 57.0 ± 14.1 years in the HF, with a predominance of male patients in both groups. More than half of patients had some primary education and more than 90% reported a monthly income of less than five minimum wages. In ACS, the diagnosis of ACS with ST segment elevation was predominant (66.3%), and 2.9% of patients died. In HF, the most common etiology was Chagas disease (25.8%), and 17.9% died. Analysis of the performance indicators revealed an adherence rate higher than 85% to nine of the 12 indicators.

**Conclusion:**

Quality programs are essential for improvement of quality of care. Performance indicators pointed to a good adherence to the SBC guidelines, mainly in the ACS arm.

## Introduction

Cardiovascular diseases (CVD) are the main cause of death in the world and one of the five leading causes of years of healthy life lost.^1^In Brazil, acute coronary syndrome (ACS) and heart failure (HF) are the main causes of death and hospitalization, respectively.^2^

National and international organizations have developed and spread recommendations by clinical guidelines to help healthcare professionals in the management of CVD.^3 - 5^Despite the vast number of publications of proven effective therapies, the adherence to guidelines’ recommendations is still lower than ideal,^6^particularly in Brazilian hospitals, as reported in the ACCEPT,^7^BRACE,^8^and BREATHE^9^registries.

In this context, programs for healthcare quality improvement have been developed trying to assure an adequate care for CVD patients. In the United States, the Get With The Guidelines (GWTG) initiative was created by the American Heart Association (AHA) and the American Stroke Association to improve the care of these patients.^10^In Brazil, the Brazilian Society of Cardiology (SBC) and the Ministry of Health, together with the AHA and with the collaboration of Hcor (Hospital do Coração) in Sao Paulo, created the Good Practices in Cardiology program (GPC program). This program is aimed at evaluating the rates of adherence to the recommendations of the SBC guidelines and results of the implementation of a quality program regarding clinical outcomes of hospitalized patients with CVD.^11^

Then, the present study aimed to characterize hospitalization data of patients with ACS and HF, participants of the GPC program, in a Brazilian public tertiary hospital, and to evaluate the rates of adherence to evidence-based therapies established by the SBC and defined as performance indicators.

## Methods

This was a descriptive cross-sectional study of analysis of data of patients hospitalized in the General Hospital of the Federal University of Minas Gerais (UFMG) between May 2016 and September 2019 with the primary diagnosis of HF or ACS. Design and basis of the program have been previously published in detail,^11^and the arms conducted in this hospital were approved by the ethics committee of the UFMG (approval number 1.487.029, April 11, 2016).

### Population

Patients aged 18 years or older, admitted with the primary diagnosis of ACS (ICD-10 I20.0 - I21.9 and I22.0 - I22.9) or HF (ICD-10 codes I50.0; I50.1 or I50.9), regardless of previous history of these conditions, were included in the study. Exclusion criteria were:

(A) Patients with ACS secondary to elective myocardial revascularization (elective percutaneous coronary intervention or myocardial revascularization surgery) or to major, non-cardiac surgeries during hospitalization;

(B) Patients with a history of HF, admitted to the emergency department with a confirmed diagnosis of dyspnea for other causes. Patients admitted for HF, patients with planned transfer to another institution and those with planned hospitalization for less than 24 hours were also excluded.

### Data collection

After screening for eligibility criteria, patients were invited to participate in the GPC program during their hospitalization, and all those willing to participate signed an informed consent form. Data were collected from the medical records and through a structured in-person interview conducted by trained investigators, using specific forms developed to the GPC program.^11^

### Outcomes

The primary outcome of the GPC program was adherence rates, by the care staff, to the performance indicators, which are quality metrics determined by class I recommendations of the SBC and AHA guidelines, described in detail in the article by Thomas et al.^11^and in the GPC website (http://www.cardiol.br/boaspraticasclinicas/).

The performance indicators in the ACS arm were ( Supplement 1 ):

1. Early aspirin use;
2. Adequate reperfusion therapy (door-to-needle time and door-to-balloon time);
3. Aspirin at discharge;
4. Angiotensin converting enzyme inhibitors (ACEI) or angiotensin receptor blocker (ARB) at discharge of patients with left ventricular ejection fraction (LVEF) < 45%;
5. Beta-blocker at discharge;
6. Statins at discharge;
7. Blood pressure control at discharge;
8. Tobacco cessation counseling for active smokers at discharge.

The performance indicators in the HF arm were ( Supplement 1 ):

1. Left ventricular function assessment by echocardiogram;
2. ACEI or ARB at discharge;
3. Beta-blocker at discharge;
4. Spironolactone at discharge;
5. Post-discharge visit (outpatient follow-up scheduled at discharge)

For the GPC program, a minimum of 85% adherence rate to the performance indicators above described was established, based on previous results of the GWTG program.^12^

### Statistical analysis

Since this was a descriptive observational study of a convenience sample, the sample size was not calculated. Continuous variables were described as mean and standard deviation or as median and interquartile range (Q1, Q3), according to results of the Shapiro-Wilk normality test. The level of significance was set at 5%. Categorical variables were expressed as proportions. Only valid data were included in the analyses. Thus, patients with missing data and patients with contraindications to the medications that composed the performance indicators, recorded in the medical records. Data were analyzed using the SPSS software version 20.1 (IBM Corp, Armonk, NY, US).

## Results

A total of 1036 patients were included, 763 with diagnosis of ACS (ACS arm) and 273 patients with diagnosis of HF (HF arm). Mean age of participants was 59.8 ± 12.0 years in the ACS arm and 57.0 ± 14.1 years in the HF arm, with predominance of male patients in both.

Main comorbidities of the patients were hypertension, diabetes, and dyslipidemia. In the HF arm, there was also a high proportion of patients with hypothyroidism and other underlying diseases. A considerable number of patients reported current or past smoking, especially in the ACS arm ( Table 1 ).

**Table 1:** – Characteristics of the sample at hospital admission (general data and medical history)

Variable		Acute coronary syndrome (763)	Heart failure (273)
Age, m ± sd*		59.8 ± 12.0	57.0 ± 14.1
Female sex		225/763 (29.5%)	119/273 (43.6%)
Comorbidities	Systemic arterial hypertension	497/633 (78.5%)	108/260 (41.5%)
	Diabetes Mellitus	220/633 (34.8%)	69/260 (26.5%)
	Dyslipidemia	171/633 (27.0%)	38/260 (14.6%)
	Stroke or transient ischemic attack	49/633 (7.7%)	18/260 (12.3%)
	Peripheral arterial disease	38/633 (6.0%)	17/260 (6.5%)
	Hypothyroidism	35/633 (5.5%)	41/260 (15.8%)
	Chronic pulmonary obstructive disease/Asthma	47/633 (7.4%)	29/260 (11.2%)
Previous heart disease	Coronary disease	162/633 (25.6%)	29/260 (11.2%)
	Acute myocardial infarction	119/633 (18.8%)	44/260 (16.9%)
	Heart failure	30/633 (4.7%)	236/260 (90.8%)
	Atrial fibrillation or atrial flutter	21/633 (3.3%)	99/260 (38.1%)
	Heart valve disease	8/633 (1.3%)	39/264 (15.2%)
	Chagas disease	6/633 (0.8%)	66/264 (25.8%)
Previous cardiac procedures	Coronary angioplasty	98/763 (12.8%)	31/273 (11.4%)
	Myocardial revascularization surgery	39/763 (5.1%)	8/273 (2.9%)
	Pacemaker	6/763 (0.8%)	43/273 (15.8%)
	Cardiac resynchronization	2/763 (0.3%)	4/273 (1.5%)
	Cardioverter-defibrillator	5/763 (0.7%)	39/273 (14.3%)
	Cardiac transplantation	-	9/273 (6.3%)
Smoking	Current and previous (total)	509/761 (66.8%)	139/266 (55.3%)
	Current	253/761 (33.2%)	21/266 (7.9%)
	Previous	256/761 (33.6%)	118/266 (44.4%)
	No	252/761 (33.1%)	127/266 (47.7%)

* Variable as mean ± standard deviation (m±sd)

More than half of patients included in the study have not finished elementary school and more than 90% reported a monthly income lower than five minimum wages ( Table 2 ).

**Table 2:** – Characteristics of socioeconomic variables

Variable		Acute coronary syndrome (763)	Heart failure (273)
Educational attainment	Illiterate	72/761 (9.5%)	29/268 (10.8%)
	Some primary education	372/761 (48.9%)	127/268 (47.4%)
	Complete primary education	91/761 (12.0%)	38/268 (14.2%)
	Some secondary education	67/761 (8.8%)	12/268 (4.5%)
	Complete secondary education	85/761 (11.2%)	52/268 (19.4%)
	Some higher education	34/761 (4.5%)	5/268 (1.9%)
	Complete higher education	40/761 (5.3%)	5/268 (1.9%)
Family income	≤ 1 minimum wage	192/761 (25.2%)	76/268 (28.4%)
	> 1 - ≤ 2 minimum wages	276/761 (36.3%)	131/268 (48.9%)
	> 2 - ≤ 5 minimum wages	226/761 (29.7%)	46/268 (17.2%)
	> 5 - ≤ 10 minimum wages	47/761 (6.2%)	12/268 (4.5%)
	> 10 minimum wages	20/761 (2.6%)	3/268 (1.1%)

In the ACS arm ( Table 3 ), most patients were Killip-Kimball class I-II (81.6%) and more than one third (37.4%) had recurrent chest pain in the first 24 hours of admission. Acute myocardial infarction (AMI) with ST-segment elevation was the predominant diagnosis (66.3%); 42.9% of these patients were thrombolysed, 33.8% underwent primary angioplasty and 23.3% did not undergo any reperfusion therapy.

**Table 3:** – Characteristics of patients with acute coronary syndrome at hospital admission

Type of acute coronary syndrome	Unstable angina		94/758 (12.4%)
	AMI without ST elevation		156/758 (20.6%)
	AMI with ST elevation		506/758 (66.7%)
Killip-Kimball classification	Killip I		491/755 (65.0%)
	Killip II		125/755 (16.5%)
	Killip III		55/755 (7.2%)
	Killip IV		58/755 (7.7%)
Cardiorespiratory arrest at admission			59/759 (7.8%)
Recurrence of pain in the first 24h			220/589 (37.4%)
Laboratory tests at admission*	Creatinine mg/dL – mean ± SD		0.96 ± 0.2
	Total cholesterol mg/dL - median (IIQ)		168 (140-202)
	LDL-C mg/dL - median (IQR)		98 (72-129)
	HDL-C mg/dL - median (IQR)		41 (34-49)
	Triglycerides. mg/dL - median (IQR)		112 (76-170)
Candidates for reperfusion			506/757 (67.1%)
Pharmacological reperfusion therapy	Thrombolytics		217/506 (42.9%)
	Type of thrombolytic agent	Streptokinase	1/217 (0.5%)
		Alteplase	175/217 (80.6%)
		Not documented	41/217 (18.9%)
Coronary angiography	Diagnostic coronary angiography		706/757 (93.3%)
	PCI of the culprit artery		495/756 (65.5%)
	Primary PCI		171/506 (33.8%)
	Rescue PCI		84/495 (17.0%)
Coronary angiography (site of the lesions)	No lesions		45/547 (8.6%)
	One vessel		150/547 (27.4%)
	Two vessels		145/547 (26.5%)
	Three vessels		207/547 (37.8%)
	LCA lesion		21/547 (3.8%)
	Lesion of the proximal DA		187/547 (34.2%)
Left ventricular ejection fraction, % - md (IQR)*			54 (43-62)
Myocardial revascularization surgery			43/763 (5.6%)
Number of days in intensive care – median (IQR)*			4 (3-6)

*HDL: high-density lipoprotein; LDL: low-density lipoprotein; AMI: acute myocardial infarction; PCI: percutaneous coronary intervention; LCA: left coronary artery; DA: descending aorta; SD: standard deviation; IQR: interquartile range. Variables presented as mean ± standard deviation (SD) or median and 1^*st*^and 3rd quartiles.*

In the HF arm ( Table 4 and Supplement 2 ), the most common etiologies were Chagas disease (25.8%), idiopathic disease (22.3%), ischemic heart disease (15.2%) and heart valve disease (15.2%). Most patients were NYHA functional class III-IV (76.4%), and most had a “warm and wet” hemodynamic profile (60.1%) on admission. Mean LVEF was 35.0% ± 9.0% and 50 patients (18.3%) were referred for heart transplantation during the same hospitalization. Dobutamine was used at any time during hospitalization in 53.9% of the patients, either for the “cold” profile on admission or for progression to signs of low cardiac output; intravenous furosemide was administered in bolus doses in 90.2% of patients and by continuous infusion using an infusion pump in 25.4%.

**Table 4:** – Characteristics of heart failure patients at hospital admission

Etiology of heart failure	Chagas disease	66/264 (25.8%)
	idiopathic	57/264 (22.3%)
	Ischemic	39/264 (15.2%)
	Valvular	39/264 (15.2%)
	Hypertrophic	8/264 (3.1%)
	Cardiotoxicity	5/264 (2.0%)
	Hypertensive	5/264 (2.0%)
	Others	45/264 (17.0%)
Number of hospitalizations in the last six months	0	44/184 (23.9%)
	1	40/184 (21.7%)
	2	42/184 (22.8%)
	> 2	58/184 (31.5%)
New York Heart Association (NYHA) functional class	I	3/254 (1.2%)
	II	16/254 (5.9%)
	III	70/254 (27.6%)
	IV	124/254 (48.8%)
Patient on the waiting list for heart transplantation	Yes	70/259 (27%)
	No	189/259 (73%)
Hemodynamic profile	Warm and dry	8/253 (3.2%)
	Warm and wet	152/253 (60.1%)
	Cold and wet	62/253 (24.5%)
	Cold and dry	8/253 (3.2%)
Cause of decompensation	Non-adherence to medication	23/273 (8.4%)
	Non-compliance with dietary and water intake recommendations	11/273 (4.0%)
	Uncontrolled hypertension	5/273 (1.8%)
	Infection	42/273 (15.4%)
	Arrhythmia	46/273 (16.8%)
	Decompensated renal failure	11/273 (4.0%)
	Ischemia or acute coronary syndrome	14/273 (5.1%)
Echocardiographic data *	Left ventricular ejection fraction, % - m±sd	35.0 ± 9.0
	Left atrial diameter, millimeter - m±sd	49.5 ± 9.0
Other data of hospitalization	Prophylaxis of deep vein thrombosis	237/256 (92.6%)
	Weight recordings (at least 70% of hospitalization period)	173/256 (67.8%)
Discontinuation of beta-blockers at admission or during hospitalization	Yes	81/141 (57.4%)
	No	60/141 (42.6%)

* Complete data available in the system (variable denominator) – echocardiographic data (217 patients). Variable presented as mean ± standard deviation (m±sd)

Tables 5 and 6 describe data of in-hospital mortality and hospital discharge. During hospitalization, 2.9% of patients in the ACS arm and 17.9% in HF arm died.

**Table 5:** – Characteristics of patients with acute coronary syndrome on discharge

In-hospital mortality				22/763 (2.9%)
Medications on discharge		Acetylsalicylic acid		700/733 (95.5%)
		P2Y2 Inhibitors		657/733 (89.6%)
		Anticoagulant		94/733 (12.8%)
		BB		616/657 (93.7%)
				BB contraindicated for 76/733 (10.4%)
		ACEI		439/596 (73.7%)
				ACEI contraindicated for 137/733 (18.6%)
		ARB		142/635 (22.4%)
				ARB contraindicated for 98/733 (13.4%)
		Statin		707/733 (96.5%)
		Spironolactone		78/486 (16.0%)
				Spironolactone was contraindicated for 247/733 (33.7%)
Clinical data on discharge*		SBP - mmHg, md (IQR)		113 (100-130)
		DBP - mmHg, md (IQR)		70 (60-80)
		HR. beats per minute – md (IQR)		68 (63-78)
Instructions	Smoking cessation^†^	Yes		277/340 (81.4%)
		No		3/340 (0.9%)
		Non-applicable		393/733 (53.6%)
		Not documented		60/340 (17.6%)
	Lifestyle change	Yes		670/733 (91.4%)
		No		16/733 (2.2%)
		Not documented		46/733 (6.3%)
	Weight control	Yes		546/710 (76.9%)
		No		24/710 (3.3%)
		Non-applicable		23/733 (3.1%)
		Not documented		140/710 (19.7%)
	Physical exercise	Yes		660/733 (90.0%)
		No		17/733 (2.3%)
		Not documented		56/733 (7.7%)
	Referred for rehabilitation^†^	Yes		615/721 (85.2%)
		No		32/721 (4.4%)
		Non-applicable		12/733 (1.6%)
		Not documented		74/721 (10.3%)
	Use of prescribed medications	Yes		677/733 (92.4%)
		No		9/733 (1.2%)
		Not documented		47/733 (6.4%)
	Outpatient follow-up	Yes		717/733 (97.8%)
		No		4/733 (0.5%)
		Non-applicable		0/733 (0.0%)
		Not documented		12/733 (1.7%)
	Anticoagulant therapy^†^		Yes	85/114 (74.6%)
			No	14/114 (12.3%)
			Non-applicable	619/733 (84.4%)
			Not documented	15/114 (13.1%)

BB: beta-blocker; ACEI: angiotensin converting enzyme inhibitors; ARB: angiotensin receptor blocker; HR: heart rate; DBP: diastolic blood pressure; SBP: systolic blood pressure. * Variables as median and 1st and 3rd quartiles [md (IQR)]; † Calculated based on patients to whom this therapy is indicated.

**Table 6:** – Characteristics of patients with heart failure on discharge

In-hospital mortality				49/273 (17.9%)
		Anticoagulant		93/206 (45.1%)
		BB		136/145 (93.8%)
				BB contraindicated for 61/206 (29.6%)
		ACEI		72/128 (56.2%)
				ACEI contraindicated for 78/206 (37.9%)
		ARB		48/145 (33.1%)
				ARB contraindicated for 61/206 (29.6%)
		Hydralazine		27/206 (13.1%)
		Nitrate		23/206 (11.2%)
		Antiarrhythmics		30/206 (14.6%)
		Digoxin		26/206 (12.6%)
		Loop diuretics		156/206 (75.7%)
		Thiazide diuretics		26/206 (12.6%)
		Spironolactone		88/124 (70.9%)
				Spironolactone contraindicated for 82/206 (39.8%)
Clinical data on discharge*		SBP, mmHg – md (IQR)		107.1 ± 18.5
		DBP, mmHg – md (IQR)		67.3 ± 12.4
		HR, beats per minute – md (IQR)		77.9 ± 14.2
Instructions	Smoking cessation^†^	Yes		10/27 (37.0%)
		No		17/27 (63.0%)
		Non-applicable		180/207 (87.0%)
		Not documented		0/27 (0.0%)
	Weight control	Yes		122/207 (58.9%)
		No		8/207 (3.9%)
		Not documented		77/207 (37.2%)
	Physical exercise	Yes		119/206 (57.8%)
		No		16/206 (7.8%)
		Non-applicable		71/206 (34.5%)
	Referred for rehabilitation^†^	Not documented		138/196 (70.4%)
		Yes		5/196 (2.6%)
		No		11/207 (5.3%)
		Not documented		53/196 (27.0%)
	Use of prescribed medications	Yes		180/206 (87.4%)
		No		0/206 (0.0%)
		Non-applicable		26/206 (12.6%)
	Counseling in case of worsening of symptoms	Yes		141/206 (68.4%)
		No		3/206 (1.4%)
		Not documented		62/206 (30.1%)
	Vaccination counseling (Influenza and Pneumococcal)		Yes	57/207 (27.5%)
			No	21/207 (10.1%)
			Not documented	129/207 (62.3%)
	Outpatient follow-up		Yes	202/207 (97.6%)
			No	5/207 (2.4%)
			Not documented	4/207 (1.9%)
	Anticoagulant therapy^†^		Yes	53/63 (84.1%)
			No	1/63 (1.6%)
			Non applicable	77/140 (55.0%)
			Not documented	9/63 (14.3%)

BB: beta-blocker; ACEI: angiotensin converting enzyme inhibitors; ARB: angiotensin receptor blocker; HR: heart rate; DBP: diastolic blood pressure; SBP: systolic blood pressure. * Variables as median and 1st and 3rd quartiles [md (IQR)]. † Calculated based on patients to whom this therapy is indicated.

Chart 1 describes the performance indicators analyzed in the study. It is worth noting that the indicator “adequate reperfusion therapy (door-to-needle time and door-to-balloon time” was not evaluated, since public health users from the whole metropolitan area with the diagnosis of ACS can be referred for coronary angiography at the General Hospital, which made it difficult to determine these times. Adherence rates were above 85% for six of the seven indicators analyzed In the ACS arm and for only three of the five indicators in the HF arm. Central Illustration summarizes the main findings of the study.

**Chart 1:** – Performance indicators on discharge

	Performance indicators	
Acute coronary syndrome *	Early aspirin use	739/756 (99.1%)
	Aspirin at discharge	700/733 (95.5%)
	ACEI or ARB at discharge	581/635 (91.5%)
	Beta-blocker at discharge	616/657 (93.7%)
	Statin at discharge	707/733 (96.5%)
	Blood pressure control	489/570 (85.8%)
	Tobacco cessation counseling	277/340 (81.5%)
Heart failure	Left ventricular function assessment	242/243 (99.6%)
	ACEI or ARB at discharge	120/145 (82.7%)
	Beta-blocker at discharge	136/145 (93.8%)
	Spironolactone at discharge	88/124 (70.9%)
	Post-discharge outpatient visit	202/207 (97.6%)

*ACEI: Angiotensin converting enzyme inhibitors; ARB: angiotensin receptor blocker. The indicator “adequate reperfusion therapy” was not evaluated since this hospital is a referral center for primary angioplasty, and all patients of this sample already had the diagnosis of acute coronary syndrome when referred for this hospital.*

**Central Illustration:** : Cardiovascular Statistics from the Good Practices in Cardiology Program – Data from a Brazilian Tertiary Public Hospital

*HF: heart failure; ACEI: angiotensin converting enzyme inhibitor; ARB: angiotensin receptor blocker; SBC: Sociedade Brasileira de Cardiologia (Brazilian Society of Cardiology).*

## Discussion

Data of the present study characterize patients hospitalized for ACS and HF in a public tertiary hospital, which can provide all lines of treatment for cardiac patients who attend public health institutions. Although patients had a low income, high proportion of partial illiteracy, and highly complex conditions, it did not preclude an adequate adherence to evidence-based therapies established by the SBC, particularly in the system of care of ACS.

In Brazil, the SBC led the construction of clinical registries of CVD, including the national registry of ACS (the ACCEPT registry).^7^Preliminary data indicate a high in-hospital mortality rate as well as low prescription rates of therapies proven effective in hospitalized patients. These data are in agreement with the BRACE (Brazilian Registry on Acute Coronary Syndromes).^8^

As compared with the ACCEPT registry^7^and the BRACE,^8^hospitalized patients with ACS in the UFMG General Hospital are slightly younger, but do have a similar profile in terms of comorbidities, particularly high rates of hypertension, diabetes and smoking. However, two remarkable differences stand out – first, there was a predominance of AMI with ST segment elevation, as the general hospital is a referral center for primary angioplasty; second, the performance indicators analyzed in the ACS arm are quantitively better than those analyzed on patient discharge in the BRACE.

Importantly, in the city of Belo Horizonte, aiming at improving the access of patients covered by the Brazilian Unified Health System to ACS treatment, a system of care for AMI was implemented between 2010 and 2011.^13^In this regard, the active participation of the general hospital has always stood out by the involvement of its telehealth center, coronary unit, division of cardiovascular surgery, cardiovascular hemodynamics, ward beds dedicated to these patients, in addition to the outpatient center for post-discharge follow-up care. Therefore, this system of care had already been monitored in the hospital and, based on previously published data,^14^the continuing education process has allowed the results of the performance indicators to be presented in the current study.

Another national registry, also held by the SBC, is the BREATHE,^9^of the system of care for HF, which similarly to ACS, has reported a low prescription rate of medications based on the best evidence for this disease. When data of the present study are compared with those of the BREATHE registry, patients of the UFMG General Hospital are different in terms of the main etiologies of HF and higher clinical complexity. This is characterized by the presence of many patients assessed for eligibility or referred for cardiac transplantation, by the frequent use of inotropes and vasodilators, and high in-hospital mortality rates. Analysis of the performance indicators in this arm indicates the need for improvements in discharge prescription and in the instructions to patients with HF.

Following the world trend, public health costs have increased in Brazil,^15^and a series of initiatives to improve system efficacy, particularly those aiming at improving the quality of care provided to patients with CVD can be implemented.^16^Based on this, the SBC created the GPC program^11^and the UFMG General Hospital implemented this program in our institution. In the same line, projects including text messaging by cell phone to improve the control of cardiovascular risk factors after discharge were also developed in the hospital.^17 , 18^Data of the GWTG program are robust and show that adherence to the guidelines have drastically increased over time,^19^resulting in clinical benefits to the patients seen in the institutions participants of this program in the United States.^20^The SBC is engaged in providing the best care possible to all patients with CVD in Brazil.

Data of the GPC program carried out in the UFMG General Hospital described in the present study have important limitations. First, the number of missing data of some variables was high, which may have influenced the analysis of results. Second, since this study required the consent of the patient or a family member, in-hospital mortality may be underestimated, since patients with severe clinical conditions that had died before the consent form was signed may have not been included in the sample. Third, data collection could not have been consecutive during the whole period of analysis, either because of problems related to the staff involved in the GPC program or because of short periods of program interruption in the country. Finally, results of 30-day or six-month follow-up after discharge could not be presented due to the high amount of incomplete data.

However, the presentation of nationwide, reliable and comprehensive data on CVD is a must to overcome inequalities and to provide the best care for cardiovascular disease patients. This study summarizes important information on ACS and HF in patients hospitalized in a public tertiary hospital in Brazil and helps to fill a knowledge gap in the literature about the subject, an important step in the planning of health politics in Brazil.

## Conclusion

The adherence to quality programs like the GPC program is crucial to improve care for patients hospitalized for ACS and HF. Analysis of the performance indicators revealed a good adherence to the SBC guidelines, particularly to those in the system of care for ACS.

## * Supplemental Materials

For additional information Supplemental Material 1, please click here .

For additional information Supplemental Material 2, please click here .
